# Effects of Curcumin/Turmeric Supplementation on Kidney Function in Individuals With Diabetes: A Systematic Review and Meta‐Analysis of Randomised Controlled Trials

**DOI:** 10.1002/edm2.70189

**Published:** 2026-02-28

**Authors:** Hossein Bahari, Zahra Asadi

**Affiliations:** ^1^ Department of Nutrition, Faculty of Medicine Mashhad University of Medical Sciences Mashhad Iran; ^2^ Student Research Committee Shiraz University of Medical Sciences Shiraz Iran

**Keywords:** creatinine, curcumin, diabetes, kidney function, meta‐analysis, systematic review, turmeric, urea

## Abstract

**Background:**

Curcumin and turmeric are widely studied for their potential renoprotective effects, but evidence regarding their impact on kidney function in individuals with diabetes remains inconsistent.

**Objective:**

To systematically evaluate the effects of curcumin/turmeric supplementation on kidney function parameters in subjects with diabetes.

**Methods:**

PubMed, Web of Science and Scopus were searched from inception until August 2025. Randomised controlled trials (RCTs) assessing the effects of curcumin/turmeric on serum creatinine, blood urea nitrogen (BUN), uric acid, urea and albumin in diabetic populations were included. Data were pooled using random‐effects models and reported as weighted mean differences (WMDs) with 95% confidence intervals (CIs). Subgroup, sensitivity, and meta‐regression analyses were conducted. Risk of bias was assessed using the Cochrane RoB 2 tool, and evidence certainty was evaluated via GRADE.

**Results:**

Twelve RCTs (*n* = 1303 participants) were included. Curcumin/turmeric supplementation did not significantly affect creatinine (WMD: −0.01 mg/dL, 95% CI: −0.03 to 0.01, *p* = 0.519) or BUN (WMD: −0.41 mg/dL, 95% CI: −3.15 to 2.33, *p* = 0.769). A trend toward reduction in uric acid was observed (WMD: −1.46 mg/dL, 95% CI: −2.93 to 0.02, *p* = 0.053). However, urea levels were significantly reduced (WMD: −2.53 mg/dL, 95% CI: −4.09 to −0.96, *p* = 0.002). No significant change was found in albumin (WMD: 0.07 g/dL, 95% CI: −0.02 to 0.17, *p* = 0.133). Subgroup analyses showed no significant moderating effects. Publication bias was detected for creatinine (Egger's *p* = 0.028). The certainty of evidence was moderate for creatinine, urea, and albumin, and low for BUN and uric acid.

**Conclusion:**

Curcumin/turmeric supplementation significantly reduces urea levels but does not significantly affect creatinine, BUN, uric acid or albumin in individuals with diabetes. Further high‐quality, long‐term RCTs are needed to clarify its renoprotective potential and optimal dosing.

## Graphical abstract

This meta‐analysis of 12 randomised controlled trials (*n* = 1303) found that curcumin/turmeric supplementation significantly reduces serum urea levels in individuals with diabetes, but does not significantly affect creatinine, BUN, uric acid or albumin. The findings suggest a potential tubular benefit, though glomerular markers remain unchanged. Further long‐term trials are needed to clarify its renoprotective role.

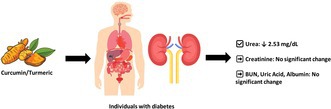

## 1 Introduction

Diabetes mellitus represents a major global health burden, with its prevalence projected to rise to 700 million cases by 2045 [1]. Among its most serious complications is diabetic kidney disease (DKD), which affects approximately 40% of individuals with diabetes and remains a leading cause of end‐stage renal disease worldwide [2]. The pathophysiology of DKD is multifactorial, involving hyperglycaemia‐induced oxidative stress, chronic inflammation, and advanced glycation end‐product accumulation, all contributing to glomerular and tubular injury [3, 4]. Despite advances in pharmacological management with agents such as sodium‐glucose cotransporter‐2 inhibitors and glucagon‐like peptide‐1 receptor agonists, the residual risk of kidney function decline persists, underscoring the need for adjunctive therapeutic strategies [5, 6].

In recent years, there has been growing interest in the potential of nutraceuticals and phytochemicals as complementary interventions to mitigate diabetic complications [7]. Curcumin, the principal bioactive polyphenol derived from turmeric ( *Curcuma longa* ), has attracted significant attention due to its potent anti‐inflammatory, antioxidant, and antifibrotic properties [8, 9, 10, 11]. Experimental studies have demonstrated that curcumin attenuates renal injury in diabetic models by modulating the nuclear factor kappa B (NF‐κB) pathway, reducing transforming growth factor‐beta (TGF‐β) expression, and inhibiting the formation of advanced glycation end‐products [12, 13]. These mechanistic insights provide a strong rationale for its potential renoprotective effects in clinical settings.

Several randomised controlled trials (RCTs) have investigated the impact of curcumin or turmeric supplementation on kidney function parameters, such as serum creatinine, blood urea nitrogen (BUN), uric acid, urea, and albumin, in individuals with diabetes [14, 15, 16, 17]. However, findings have been inconsistent, with some studies reporting beneficial effects on renal biomarkers [18, 19], while others show no significant changes [20, 21]. These discrepancies may arise from variations in study design, sample characteristics, curcumin formulation, dosage and treatment duration. Prior meta‐analyses have sought to clarify these effects, with some focusing on patients with established DKD [22] and others on broader diabetic populations [23]. However, no comprehensive quantitative synthesis has yet integrated the full spectrum of kidney function biomarkers (creatinine, BUN, uric acid, urea and albumin) across diverse diabetic subgroups to clarify the overall efficacy and potential moderating factors.

To address this gap, we conducted a systematic review and meta‐analysis of RCTs to evaluate the effects of curcumin/turmeric supplementation on kidney function in subjects with diabetes, irrespective of baseline kidney status. Our analysis also explores the influence of key covariates, including baseline kidney function, intervention dose, duration, and formulation type, through subgroup and meta‐regression analyses. By synthesising the current clinical evidence, this study aims to provide a clearer and more nuanced understanding of the therapeutic potential of curcumin/turmeric in diabetic kidney health and to inform future research and clinical practice.

## 2 Methods

### 2.1 Study Design and Registration

This systematic review and meta‐analysis was conducted in accordance with the Preferred Reporting Items for Systematic Reviews and Meta‐Analyses (PRISMA 2020) guidelines [24]. The study protocol was prospectively registered with the International Prospective Register of Systematic Reviews (PROSPERO) under registration number CRD420261279897.

### 2.2 Eligibility Criteria

Studies were selected based on the following criteria:

- Participants: adults (≥ 18 years) human subjects with a diagnosis of type 1 or type 2 diabetes mellitus, with or without established kidney disease
- Intervention: oral supplementation with curcumin, turmeric, or any curcuminoid‐based formulation, regardless of dosage, duration or formulation type (e.g., unformulated curcumin, piperine‐enhanced, nano‐curcumin, etc.).
- Comparator: placebo or active control.
- Outcomes: primary outcomes included changes in serum kidney function parameters: serum creatinine, BUN, uric acid, urea and albumin. Studies reporting at least one of these outcomes were included. BUN and urea are related measures of nitrogen waste; BUN reflects urea nitrogen content. Studies reporting either were included, and analyses were conducted separately.
- Study design: randomised controlled trials (RCTs) published in English.

Exclusion criteria were: non‐randomised studies, observational studies, reviews, conference abstracts without full text, studies on non‐diabetic populations, and trials using curcumin as part of a multi‐component supplement without an isolated curcumin arm.

### 2.3 Information Sources and Search Strategy

A comprehensive literature search was performed across three electronic databases: PubMed, Web of Science, and Scopus, from inception until August 31, 2025. The search strategy was developed in consultation with a medical librarian and combined Medical Subject Headings (MeSH) terms and keywords related to *curcumin*, *turmeric*, *diabetes* and *kidney function*. The full PubMed search strategy is provided in Table S1. In addition, reference lists of relevant reviews and included articles were manually screened for additional eligible studies.

### 2.4 Study Selection and Data Extraction

Two independent reviewers (H.B. and Z.A.) screened titles, abstracts, and full‐text articles using the web‐based tool Rayyan. Disagreements were resolved through discussion and consensus. A standardised, piloted data extraction form was used to collect the following information:

- Study characteristics: first author, publication year, country, study design, blinding method.
- Participant details: sample size, age, sex, baseline kidney function, diabetes type and duration, comorbidities.
- Intervention data: type of curcumin/turmeric formulation, dosage (mg/day), duration of supplementation (weeks), control type.
- Outcome data: baseline and post‐intervention means and standard deviations (SDs) for each kidney parameter. If not reported, values were estimated from figures, calculated using established formulas, or requested from authors.

### 2.5 Risk of Bias Assessment

The Cochrane Risk of Bias 2 (RoB 2) tool for randomised trials was used to assess the methodological quality of each included study [25]. Each trial was evaluated across five domains: (1) bias arising from the randomization process, (2) bias due to deviations from intended interventions, (3) bias due to missing outcome data, (4) bias in measurement of the outcome, and (5) bias in selection of the reported result. Two reviewers independently rated each domain as ‘low risk’, ‘some concerns’ or ‘high risk’, with disagreements resolved by consensus.

### 2.6 Data Synthesis and Statistical Analysis

The meta‐analysis was performed using STATA software version 17 (Stata Corp, College Station, TX, USA). Continuous outcome data were pooled as weighted mean differences (WMDs) with 95% confidence intervals (CIs) using a random‐effects model (DerSimonian and Laird method) to account for expected clinical and methodological heterogeneity [26]. The *I* ^2^ statistic and Cochran's *Q* test (*p* < 0.10) were used to quantify and assess statistical heterogeneity, with *I* ^2^ values of 25%, 50% and 75% indicating low, moderate and high heterogeneity, respectively [27].

#### 2.6.1 Subgroup and Sensitivity Analyses

Pre‐specified subgroup analyses were conducted for the primary outcome (creatinine) to explore potential sources of heterogeneity, based on:

- Baseline creatinine level (≤ 1.1 vs. > 1.1 mg/dL).
- Trial duration (< 12 vs. ≥ 12 weeks).
- Intervention dose (< 1 vs. ≥ 1 g/day).
- Formulation type (unformulated, piperine‐enhanced, formulated/nano‐curcumin).
- Health status (diabetes without kidney disease vs. diabetes with established kidney disorders).

Between‐subgroup differences were assessed using meta‐regression for continuous moderators (dose, duration) and subgroup interaction tests for categorical variables. Sensitivity analyses were performed using the leave‐one‐out method to evaluate the robustness of pooled estimates [28].

The cutoff of 1.1 mg/dL for baseline creatinine was based on the upper limit of the normal reference range for serum creatinine in adults, as commonly applied in clinical practice and prior nephrology research [29, 30].

#### 2.6.2 Publication Bias

Publication bias was assessed visually using funnel plots and statistically using Egger's linear regression test and Begg's rank correlation test when ≥ 10 studies were available for an outcome [31, 32].

#### 2.6.3 Meta‐Regression and Dose–Response Analysis

To further examine the influence of intervention characteristics on creatinine levels, random‐effects meta‐regression was performed using dose (mg/day) and duration (weeks) as continuous predictors. An exploratory dose–response analysis was also conducted using a one‐stage linear mixed‐effects model to estimate potential non‐linear trends.

### 2.7 Certainty of Evidence

The overall quality of evidence for each outcome was evaluated using the Grading of Recommendations Assessment, Development and Evaluation (GRADE) framework [33]. Evidence was rated as high, moderate, low, or very low based on risk of bias, inconsistency, indirectness, imprecision, and publication bias.

## 3 Results

### 3.1 Study Selection

**FIGURE 1:**
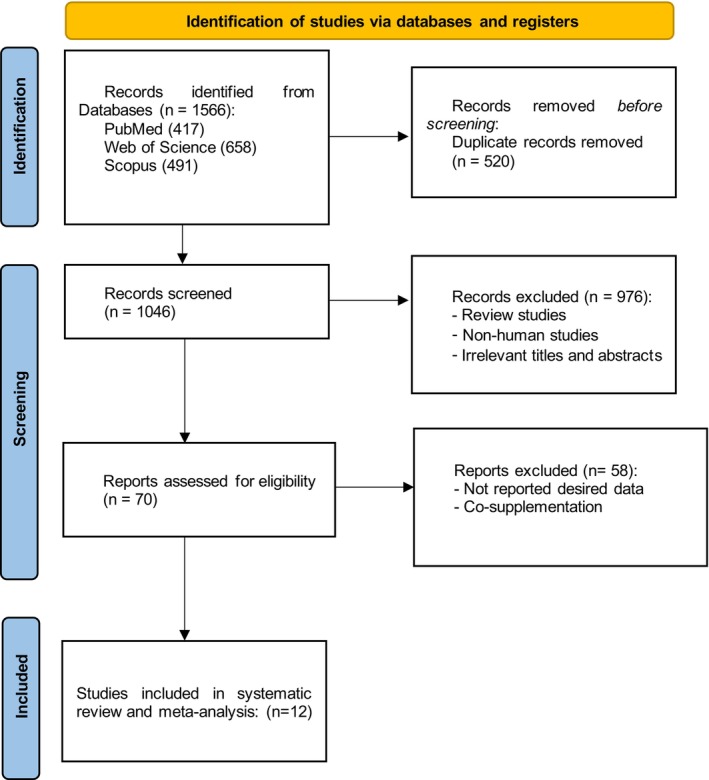
PRISMA flow chart of study selection process in the systematic review.

The PRISMA flow diagram detailing the study selection process is presented in Figure 1. A total of 1566 records were identified from three electronic databases: PubMed (*n* = 417), Web of Science (*n* = 658) and Scopus (*n* = 491). After removing 520 duplicate records, 1046 studies were screened by title and abstract. Of these, 976 were excluded as review articles, non‐human studies, or irrelevant publications. Following full‐text assessment of the remaining 70 articles, 58 were excluded due to the lack of relevant outcome data or co‐supplementation with other compounds. Ultimately, 12 randomised controlled trials met the inclusion criteria and were included in the systematic review and meta‐analysis [14, 15, 16, 17, 18, 19, 20, 21, 34, 35, 36, 37].

### 3.2 Characteristics of Included Studies

The baseline characteristics of the included studies are summarised in Table 1. The studies were published between 2011 and 2025 and conducted across six countries: Iran (*n* = 6), Thailand (*n* = 3), India (*n* = 1), Mexico (*n* = 1), and Japan (*n* = 1). A total of 1303 participants were included, with sample sizes ranging from 30 to 227. All studies were randomised and placebo‐controlled, with 10 employing a double‐blind design. The intervention duration varied from 4 to 48 weeks. The forms of curcumin/turmeric supplementation included unformulated curcumin, piperine‐enhanced formulations, nano‐curcumin, and turmeric powder, with doses ranging from 80 to 2000 mg/day. The included populations comprised individuals with type 2 diabetes (T2DM), diabetic nephropathy, diabetic proteinuric chronic kidney disease (CKD), and related metabolic conditions.

**TABLE 1:** Characteristics of included studies in meta‐analysis.

Studies	Country	Study design	Participant	Sex	Sample size		Trial duration (weeks)	Means age		Means BMI		Intervention			Main outcomes
					IG	CG		IG	CG	IG	CG	Type	Dose (mg/day)	Control group	
Khajehdehi et al. [14]	Iran	R, DB, PC, Parallel	T2DM nephropathy	M/F	20	20	8	52.9	52.6	NR	NR	Rhizome of turmeric	1500	Placebo	↔ Cr and BUN
Chuengsamarn et al. [15]	Thailand	R, DB, PC, Parallel	T2DM	M/F	107	106	24	59.1	59.5	27.1	26.8	Curcuminoids	1500	Placebo	Cr, UA. ↔ Cr; ↓ UA (*p* < 0.05).
Maithili Karpaga Selvi et al. [18]	India	Open, R, CO, Parallel	T2DM	M	30	30	4	47	46.8	23.4	24.1	Turmeric powder + metformin	2000	Metformin	Cr, Urea, and Alb. ↔ Cr; ↓ Urea (*p* < 0.05), ↔ Alb
Jiménez‐Osorio et al. [19]	Mexico	R, DB, PC, Parallel	Diabetic proteinuric CKD	M/F	28	23	8	55	56.2	29.7	27.9	Turmeric	320	Placebo	↔ Cr and Urea
Panahi et al. [16]	Iran	R, DB, PC, Parallel	T2DM	M/F	50	50	12	43	41	26	27	Curcuminoids + piperine	500	Placebo	↔ Cr
Funamoto et al. [17]	Japan	R, DB, PC, Parallel	IGT and NIDDM	M/F	15	18	24	70	69	24.9	25	Theracurmin	180	Placebo	↔ Cr and UA
Vanaie et al. [20]	Iran	R, DB, PC, Parallel	Overt diabetic nephropathy	M/F	27	19	16	59	61	NR	NR	Curcumin	1500	Placebo	↓ Cr, ↔ BUN, ↔ Alb
Shafabakhsh et al. [21]	Iran	R, DB, PC, Parallel	Patients with diabetes on haemodialysis	M/F	26	27	12	58.3	56.2	27.9	27.1	Nano‐curcumin	80	Placebo	Cr, BUN. ↔ Cr; ↓ BUN (*p* < 0.05).
Yaikwawong et al. [37]	Thailand	R, DB, PC, Parallel	Obese patients with T2DM	M/F	113	114	48	60.2	62.2	27.2	26.7	Curcumin	1500	Placebo	Cr, UA. ↔ Cr; ↓ UA (*p* < 0.05).
Amini et al. [36]	Iran	R, DB, PC, Parallel	Nonproliferative diabetic retinopathy	M/F	27	29	12	55.8	55.8	27.8	27.9	Curcumin + piperine	1000	Placebo	↓ Cr, ↔ BUN
Yaikwawong et al. [35]	Thailand	R, DB, PC, Parallel	T2DM and MASLD	M/F	39	39	48	57.3	60.2	27.2	27.5	Curcumin	1500	Placebo	↔ Cr
Mansour et al. [34]	Iran	R, DB, PC, Parallel	T2DM	M/F	41	45	16	62.3	62.6	29.5	28.1	Nano‐curcumin	80	Placebo	↔ Cr and Urea

Abbreviations: ↓, significant decrease; ↔, no significant change; Alb, albumin; BMI, Body Mass Index; BUN, blood urea nitrogen; CG, control group; CKD, chronic kidney disease; CO, controlled; Cr, creatinine; DB, double‐blinded; IG, intervention group; IGT, impaired glucose tolerance; MASLD, metabolic dysfunction‐associated steatotic liver disease; NIDDM, non‐insulin dependent diabetes mellitus; NR, not reported; PC, placebo‐controlled; R, randomised; T2DM, type 2 diabetes mellitus; UA, uric acid.

### 3.3 Risk of Bias Assessment

**FIGURE 2:**
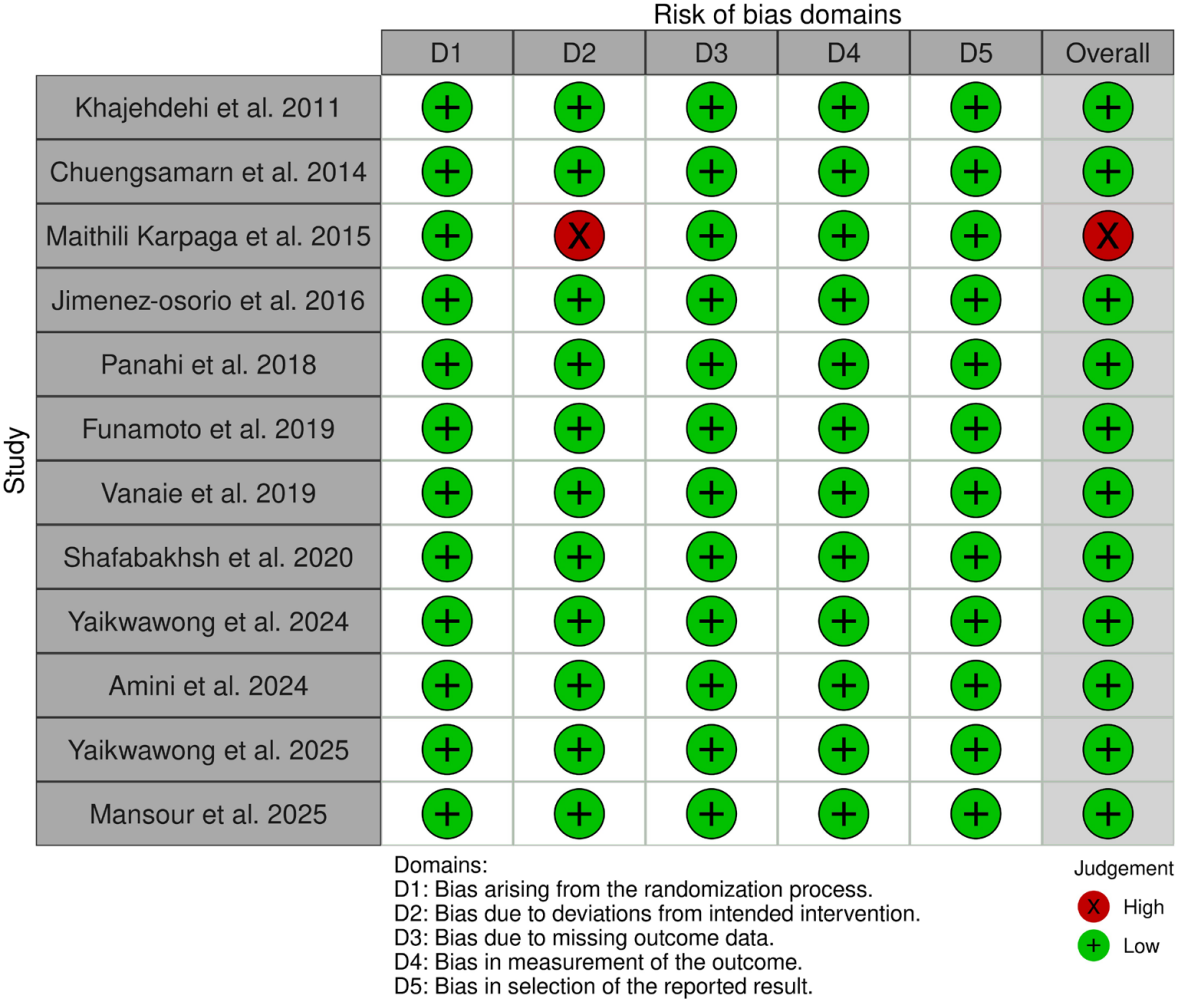
Results of risk of bias evaluation according to the Cochrane tool.

The methodological quality of the included trials was assessed using the Cochrane Risk of Bias 2 (ROB 2) tool (Figure 2). All studies were rated as low risk in domains of randomization, selection of reported results, and missing outcome data. One study [18] was rated as high risk due to deviations from intended interventions. The remaining studies were judged as low risk across all domains, resulting in an overall low risk of bias for 11 of the 12 included trials.

### 3.4 Meta‐Analysis of Kidney Function Parameters

#### 3.4.1 Creatinine

**FIGURE 3:**
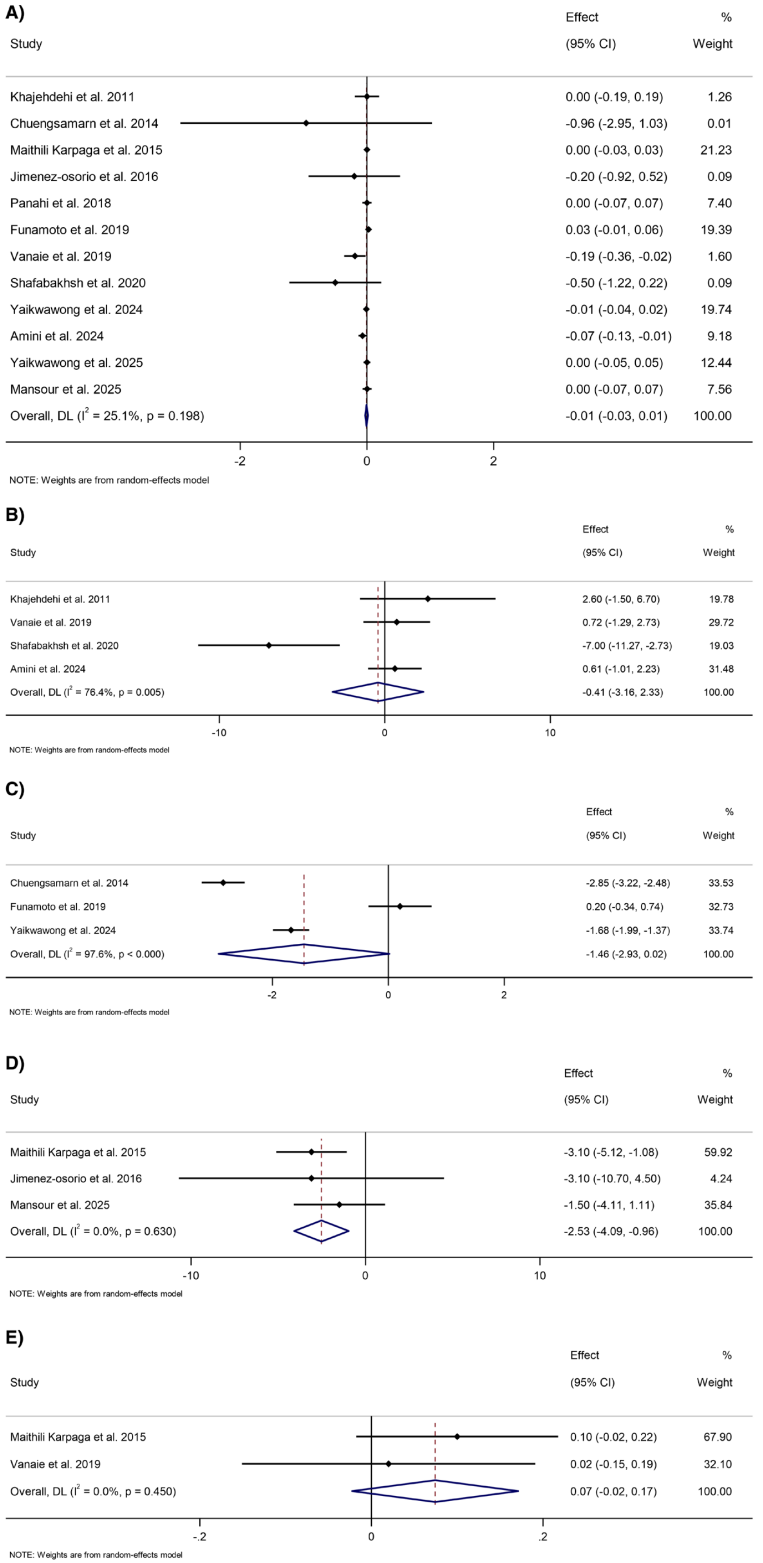
Forest plot detailing weighted mean difference and 95% confidence intervals (CIs) for the effect of curcumin/turmeric on (A) creatinine (mg/dL), (B) blood urea nitrogen (mg/dL), (C) uric acid (mg/dL), (D) urea (mg/dL), and (E) albumin (g/dL).

The overall pooled effect from 12 studies showed no significant change in serum creatinine levels following curcumin/turmeric supplementation (WMD: −0.01 mg/dL, 95% CI: −0.03 to 0.01, *p* = 0.519) (Figure 3A). Subgroup analyses based on baseline creatinine, trial duration, intervention dose, formulation type, and health status revealed no significant between‐group differences, although a trend toward reduction was observed in subgroups with baseline creatinine > 1.1 mg/dL and in participants with established kidney disorders (Table 2).

**TABLE 2:** Subgroup analyses of curcumin/turmeric on kidney function in diabetes.

	Number of effect sizes	WMD (95% CI)	*p*	Heterogeneity		
				*p* heterogeneity	*I* ^2^	*p* between sub‐groups
Curcumin intake on creatinine (mg/dL)						
Overall effect	12	−0.01 (−0.03, 0.01)	0.519	0.198	25.1%	
Baseline creatinine						
> 1.1	4	−0.12 (−0.26, 0.01)	0.08	0.35	8.5%	0.09
< 1.1	8	−0.00 (−0.02, 0.02)	0.81	0.34	11.0%	
Trial duration (weeks)						
≥ 12	9	−0.01 (−0.04, 0.02)	0.42	0.07	44.2%	0.58
< 12	3	−0.00 (−0.03, 0.03)	0.98	0.86	0.0%	
Intervention dose (g/day)						
> 1	7	−0.02 (−0.05, 0.01)	0.20	0.16	34.5%	0.09
< 1	5	0.01 (−0.01, 0.04)	0.27	0.57	0.0%	
Intervention type						
Unformulated curcumin	7	−0.01 (−0.03, 0.01)	0.51	0.42	0.0%	0.35
Piperine‐enhanced	2	−0.04 (−0.11, 0.03)	0.28	0.15	51.1%	
Formulated curcumin	3	0.02 (−0.02, 0.06)	0.39	0.31	13.0%	
Health Status						
Diabetes	8	−0.00 (−0.02, 0.02)	0.81	0.34	11.0%	0.09
Diabetes with established kidney disorders	4	−0.12 (−0.26, 0.01)	0.08	0.35	8.5%	
Curcumin intake on BUN (mg/dL)						
Overall effect	4	−0.41 (−3.15, 2.33)	0.769	0.005	76.4%	
Curcumin intake on uric acid (mg/dL)						
Overall effect	3	−1.46 (−2.93, 0.02)	0.053	< 0.001	97.6%	
Curcumin intake on urea (mg/dL)						
Overall effect	3	−2.53 (−4.09, −0.96)	**0.002**	0.63	0.0%	
Curcumin intake on albumin (g/dL)						
Overall effect	2	0.07 (−0.02, 0.17)	0.133	0.45	0.0%	

Abbreviations: BUN, blood urea nitrogen; CI, confidence interval; WMD, weighted mean differences.

#### 3.4.2 Blood Urea Nitrogen (BUN)

Four studies reported BUN outcomes, with no significant overall effect (WMD: −0.41 mg/dL, 95% CI: −3.15 to 2.33, *p* = 0.769). Substantial heterogeneity was observed (*I* ^2^ = 76.4%, *p* = 0.005) (Figure 3B).

#### 3.4.3 Uric Acid

Three studies evaluated uric acid levels, indicating a non‐significant trend toward reduction (WMD: −1.46 mg/dL, 95% CI: −2.93 to 0.02, *p* = 0.053). Heterogeneity was very high (*I* ^2^ = 97.6%, *p* < 0.001) (Figure 3C).

#### 3.4.4 Urea

A significant reduction in urea levels was observed in three studies (WMD: −2.53 mg/dL, 95% CI: −4.09 to −0.96, *p* = 0.002), with no heterogeneity (*I* ^2^ = 0%, *p* = 0.63) (Figure 3D).

#### 3.4.5 Albumin

Two studies reported albumin levels, showing no significant change (WMD: 0.07 g/dL, 95% CI: −0.02 to 0.17, *p* = 0.133) (Figure 3E).

### 3.5 Sensitivity and Subgroup Analyses

Sensitivity analysis via the leave‐one‐out method indicated that none of the pooled estimates for creatinine, BUN and albumin were substantially driven by any single study. For uric acid, the study by Funamoto et al. [17] contributed significantly to the pooled estimate (WMD when removed: −2.26, 95% CI: −3.40 to −1.11). For urea, the study by Maithili Karpaga Selvi et al. [18] influenced the overall result (WMD when removed: −1.66, 95% CI: −4.13 to 0.80). Subgroup analyses for creatinine are detailed in Table 2; none of the subgroups showed statistically significant between‐group differences.

### 3.6 Publication Bias

**FIGURE 4:**
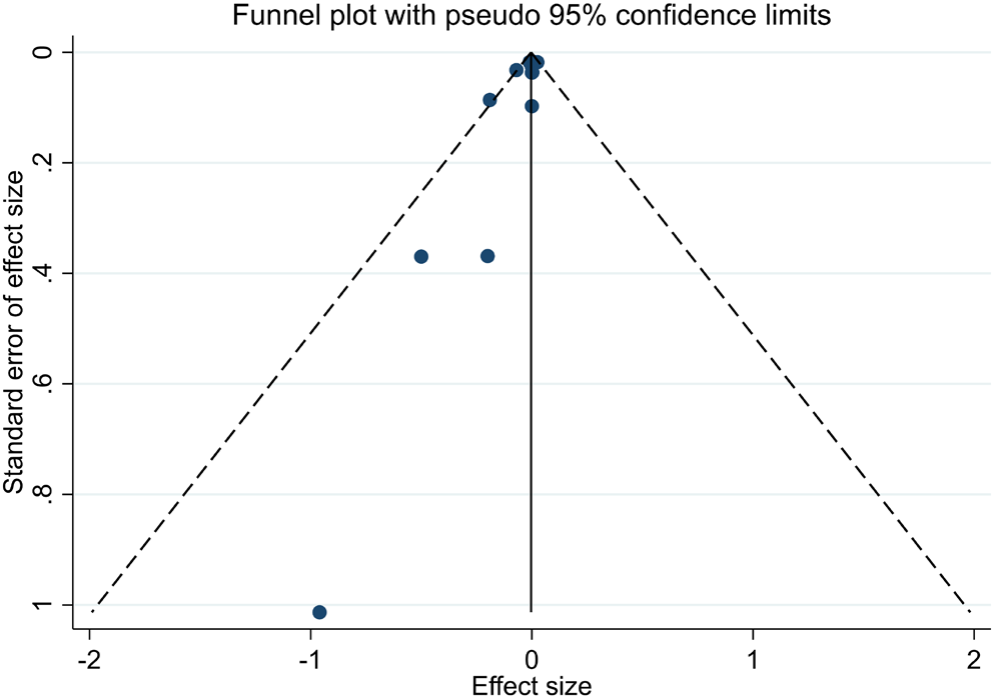
Funnel plots for the effect of curcumin/turmeric on creatinine.

Publication bias was assessed using Begg's and Egger's tests for creatinine, the only outcome with sufficient studies (*n* = 12). Both tests indicated significant publication bias (Begg's *p* = 0.047; Egger's *p* = 0.028) (Figure 4). Publication bias was not assessed for outcomes with fewer than 10 studies (e.g., urea, *n* = 3) as per methodological standards, which should be considered a limitation when interpreting these results.

### 3.7 Dose–Response and Meta‐Regression

**FIGURE 5:**
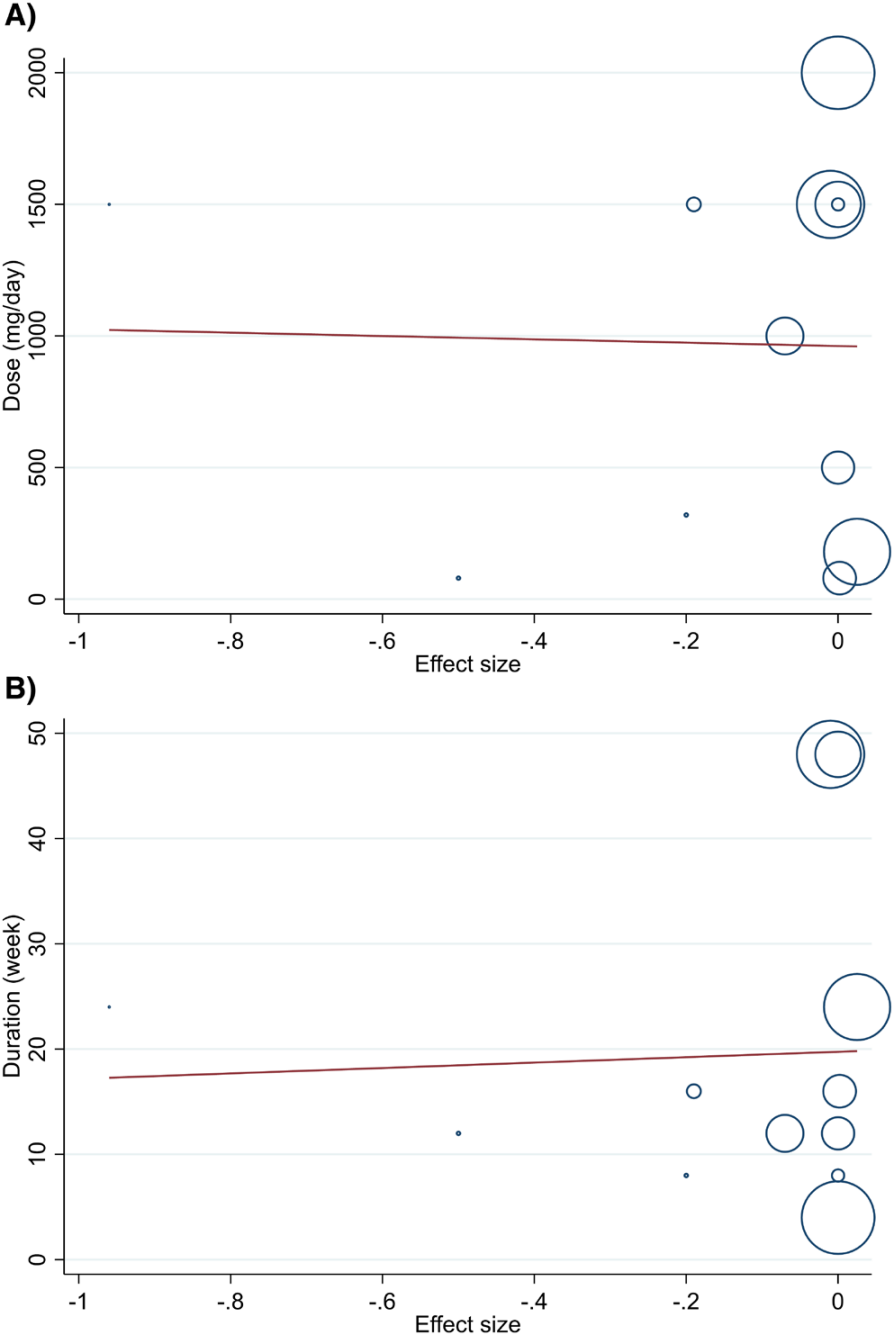
Random‐effects meta‐regression plots of the association between mean changes in creatinine (mg/dL) and (A) curcumin/turmeric dosage (mg/day) and (B) intervention duration (weeks).

**FIGURE 6:**
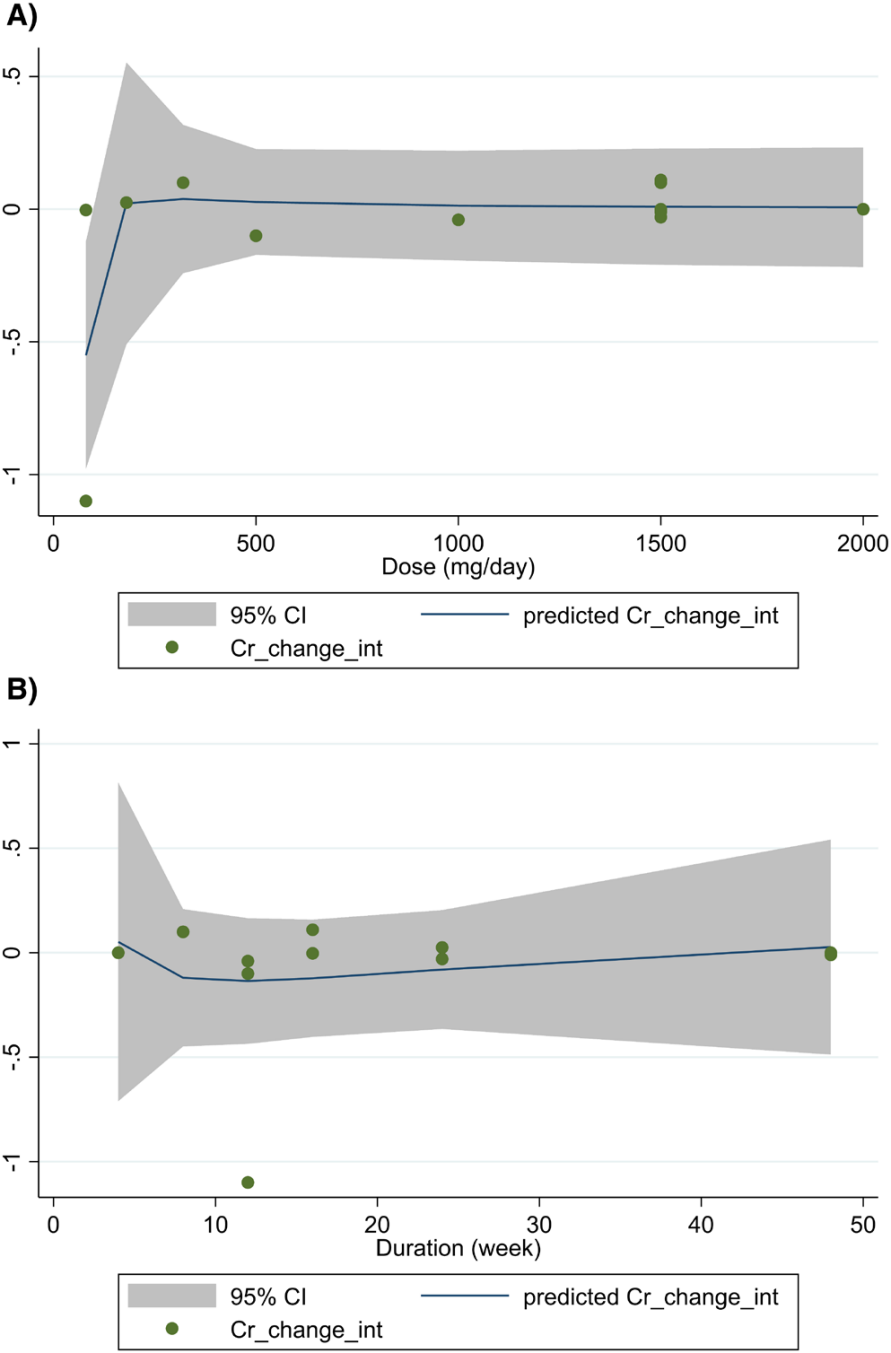
Dose–response relations between (A) dosage (mg/day) and (B) duration (weeks) of curcumin/turmeric supplementation and mean difference in creatinine (mg/dL).

Meta‐regression and dose–response analyses were conducted for creatinine to explore the influence of dose (mg/day) and trial duration (weeks). Neither dose nor duration showed a significant association with changes in creatinine levels (all *p* > 0.05) (Figures 5 and 6).

### 3.8 Certainty of Evidence

The GRADE assessment indicated that the evidence for creatinine, urea, and albumin was of moderate quality, downgraded due to publication bias or imprecision. Evidence for BUN and uric acid was rated as low quality, primarily due to serious inconsistency (high heterogeneity) and imprecision from a few included studies (Table 3).

**TABLE 3:** GRADE profile of curcumin/turmeric on kidney function in diabetes.

Outcomes	Risk of bias	Inconsistency	Indirectness	Imprecision	Publication bias	Quality of evidence
Creatinine	No serious limitation	No serious limitation	No serious limitation	No serious limitation	Serious limitation	⊕⊕⊕◯ Moderate
BUN	No serious limitation	Very serious limitation^a^	No serious limitation	Serious limitation^b^	No serious limitation	⊕⊕◯◯ Low
Uric acid	No serious limitation	Very serious limitation^a^	No serious limitation	Serious limitation^b^	No serious limitation	⊕⊕◯◯ Low
Urea	No serious limitation	No serious limitation	No serious limitation	Serious limitation^b^	No serious limitation	⊕⊕⊕◯ Moderate
Albumin	No serious limitation	No serious limitation	No serious limitation	Serious limitation^b^	No serious limitation	⊕⊕⊕◯ Moderate

^a^ There is high heterogeneity (*I* ^2^ > 75%).

^b^ Evidence was limited by the small number of included studies.

## 4 Discussion

This systematic review and meta‐analysis of 12 RCTs represents the most comprehensive quantitative synthesis to date examining the effects of curcumin/turmeric supplementation on kidney function in individuals with diabetes. The pooled results indicate that supplementation leads to a significant reduction in serum urea levels, but does not significantly alter serum creatinine, BUN, uric acid and albumin. These findings contribute nuanced insights into the potential renoprotective role of this widely studied nutraceutical.

### 4.1 Interpretation of Findings in Context

The observed reduction in urea is a noteworthy finding. Urea, while a less specific marker of glomerular function than creatinine, reflects nitrogen metabolism and tubular handling [38]. Its decrease may suggest that curcumin could influence protein catabolism or tubular function, pathways implicated in diabetic nephropathy progression, though it is important to note that urea levels are also influenced by extra‐renal factors such as dietary protein intake and hydration status [39]. This aligns with preclinical evidence where curcumin has been shown to ameliorate tubular injury and oxidative stress in diabetic rodent models [40, 41]. For instance, Wu et al. [42] demonstrated in a meta‐analysis of animal studies that curcumin administration significantly reduced BUN and serum creatinine in diabetic rodents. However, the clinical translation appears selective, as our analysis did not show significant effects on creatinine, the primary marker of glomerular filtration rate. This discrepancy may reflect different pathophysiological sensitivities or the multifactorial regulation of creatinine, which is influenced by muscle mass, age, and gender.

Our results partially contrast with the meta‐analysis by Zhao et al. [22], which reported a significant reduction in serum creatinine in patients with established DKD. This discrepancy likely stems from differences in the target population. Our analysis included diabetic individuals with and without overt kidney disease, whereas the meta‐analysis by Zhao et al. focused exclusively on patients with established DKD. This hypothesis is supported by our subgroup analysis, where a trend toward creatinine reduction was observed in participants with baseline creatinine > 1.1 mg/dL and those with established kidney disorders, although it did not reach statistical significance.

The absence of significant effects on BUN and uric acid, despite high heterogeneity, points to the complexity of these biomarkers. BUN is influenced by non‐renal factors like hydration and protein intake, while uric acid levels are affected by purine metabolism and diuretic use [43]. The considerable variability across studies in participant characteristics, comorbidities, and concomitant medications may have obscured any true treatment effect.

Importantly, the pattern of findings observed in this meta‐analysis may also be explained by the differential physiological significance of the assessed kidney biomarkers. Serum creatinine and albumin primarily reflect glomerular integrity and filtration function, whereas serum urea is more strongly influenced by nitrogen metabolism, tubular handling, and renal metabolic activity [44, 45]. Glomerular markers such as creatinine and albumin tend to change slowly and are relatively insensitive to short‐term interventions, particularly in individuals without advanced structural kidney damage [46]. In contrast, tubular and metabolic markers may respond earlier to interventions targeting oxidative stress and inflammation [47]. The selective reduction in urea observed in the present analysis, in the absence of parallel changes in creatinine or albumin, therefore supports a mechanism whereby curcumin exerts greater effects on tubular function and metabolic pathways rather than on glomerular filtration per se [48]. This distinction strengthens the biological plausibility of our findings and aligns with experimental evidence demonstrating that curcumin preferentially attenuates tubular injury and oxidative stress in diabetic kidney disease models [40, 41, 42].

### 4.2 Consideration of Intervention Characteristics

The included trials utilised a wide array of curcumin formulations (unformulated, piperine‐enhanced, nano‐curcumin) and doses (80–2000 mg/day) over durations of 4 to 48 weeks. Despite this heterogeneity, subgroup analyses did not reveal significant moderating effects of dose, duration, or formulation on the primary outcome of creatinine. This may indicate a plateau effect or, more importantly, that the absolute dose is less critical than the bioavailability of the compound. Advanced formulations like nano‐curcumin are designed to enhance bioavailability, but their comparative efficacy for renal endpoints in long‐term human studies remains to be conclusively established.

### 4.3 Mechanistic Insights and Clinical Relevance

The renoprotective mechanisms of curcumin, well‐documented in animal studies, include potent anti‐inflammatory, antioxidant, and antifibrotic actions [48]. It modulates key pathways such as NF‐κB, nuclear factor erythroid 2‐related factor 2 (Nrf2), and TGF‐β, all central to the pathogenesis of diabetic nephropathy [13, 49]. The significant reduction in urea observed in our human meta‐analysis may be a downstream reflection of these pleiotropic actions, particularly on tubular health and oxidative metabolism. However, translating robust preclinical benefits into consistent clinical outcomes for glomerular markers like creatinine appears more challenging, possibly due to the multifactorial nature of human DKD, genetic diversity and varying stages of disease at intervention [50].

From a clinical perspective, curcumin/turmeric supplementation appears safe and may offer a complementary approach to standard care, particularly for its potential metabolic benefits on glycaemia and lipids, as shown in other meta‐analyses [51, 52]. However, based on the current evidence, it should not be viewed as a substitute for foundational renoprotective therapies such as sodium‐glucose cotransporter‐2 (SGLT2) inhibitors or Renin‐angiotensin‐aldosterone system (RAAS) blockers. The urea reduction, while statistically significant, should be interpreted with caution. A reduction of −2.53 mg/dL may not translate into a clinically meaningful slowing of kidney disease progression, as a minimal clinically important difference for urea in diabetic kidney disease has not been established. Therefore, this finding requires further investigation and validation in trials with hard renal endpoints.

### 4.4 Strengths and Limitations

The key strengths of this work include a comprehensive search strategy, adherence to PRISMA guidelines, dual independent review processes, rigorous risk of bias and GRADE assessments, and extensive exploration of heterogeneity through subgroup and sensitivity analyses. Nevertheless, limitations exist. The modest number of studies for some outcomes limits the power of the analysis. Significant clinical and methodological heterogeneity is present. The high heterogeneity for BUN and uric acid may be attributed to clinical differences across studies, including variations in participant characteristics, assay methods, dietary intake, hydration status, and concomitant medications. The short‐to‐medium duration of most trials may be inadequate to evaluate changes in chronic kidney parameters. Publication bias was detected for creatinine, indicating possible unpublished null findings. Finally, safety data were inconsistently reported, preventing a full risk–benefit appraisal.

In conclusion, this meta‐analysis indicates that curcumin/turmeric supplementation significantly lowers serum urea levels in individuals with diabetes, suggesting a possible beneficial effect on nitrogen metabolism and tubular function. However, it does not demonstrate a significant impact on other key kidney function parameters, including serum creatinine, BUN, uric acid, or albumin. The certainty of evidence varies from moderate to low across outcomes.

### 4.5 Implications for Practice and Research

For clinical practice, curcumin may be considered a generally safe complementary agent within a holistic management plan for diabetes. However, its use specifically for the purpose of improving kidney function, particularly glomerular filtration, is not strongly supported by current evidence. Patients with established diabetic kidney disease might represent a subgroup that could benefit more, but this requires confirmation.

Future research should prioritise large‐scale, long‐term, rigorously designed RCTs that:

- Stratify participants by stage of diabetic kidney disease.
- Employ standardised, bioavailable curcumin formulations.
- Utilise comprehensive renal endpoints, including measured GFR, cystatin C, and urinary albumin excretion, over extended follow‐up periods.
- Incorporate mechanistic biomarkers to link clinical effects with curcumin's known pharmacological pathways.
- Systematically document safety and tolerability.

Until such high‐quality evidence is available, the potential of curcumin as a renoprotective agent in diabetes remains an encouraging but not yet fully substantiated prospect.

## Author Contributions

**Hossein Bahari:** conceptualization, investigation, writing – original draft, writing – review and editing, validation, methodology, software, formal analysis, project administration. **Zahra Asadi:** investigation, data curation, writing – original draft, writing – review and editing.

## Funding

The authors have nothing to report.

## Conflicts of Interest

The authors declare no conflicts of interest.

## Supporting information


**Table S1:** Search strategy.

## Data Availability

The data that support the findings of this study are available from the corresponding author upon reasonable request.
